# Supplementary material for: Safety, Feasibility and Efficacy of Lokomat® and Armeo®Spring Training in Deconditioned Paediatric, Adolescent and Young Adult Cancer Patients
Source: Cancers (Basel). 2023 Feb 16;15(4):1250. doi: 10.3390/cancers15041250 (PMC9954270; doi:10.3390/cancers15041250)
Supplement: Supplementary file 1 [file cancers-15-01250-s001.zip › Supplementary File S1 Safety Checklist.pdf]

Supplementary File S1: Safety Checklist

Safety Checklist for Youth Cancer Service / WCH Haem Onc Rehabilitation Robotics Trial

This list must be reviewed and documented prior to *every* Robotics session
Please phone Dr Michael Osborn (0409677321) for medical clearance prior to every session

Participant Name: Date of Birth:

Since your last Robotics session have you experienced:

1.	Fever or infection	YES	NO
2.	Anaemia (low haemoglobin or red blood cells)	YES	NO
3.	Light headedness or faints	YES	NO
4.	Bleeding or bruising for no reason	YES	NO
5.	Chest pain	YES	NO
6.	Shortness of breath or other lung problems (eg asthma)	YES	NO
7.	Any new pain	YES	NO
8.	Broken bones	YES	NO
9.	Leg or ankle swelling	YES	NO
10.	Seizures	YES	NO
11.	Skin sores, ulcers or rashes	YES	NO

Since your last Robotics session have you had any:

1.	Hospital admissions	YES	NO
2.	Emergency Department visits	YES	NO
3.	New medical problems	YES	NO
4.	Changes to medications (ie new medicines or dose changes)	YES	NO
5.	Operations	YES	NO
6.	Blood transfusions (red cells, platelets, other)	YES	NO

Are you pregnant? YES NO

If the answer is "yes" to any of these questions, record the details on the next page

Has the Medical Officer agreed this person can proceed today? YES NO

Completed by:

Name: ____________________ **Date:** ____________________

Signature: ____________________ **Designation:** ____________________

THIS DOCUMENT MUST BE STORED IN THE PATIENT'S RESEARCH FILE

Supplementary File S1: Safety Checklist

Safety Checklist for Youth Cancer Service / WCH Haem Onc Rehabilitation Robotics Trial

Details of any safety concerns noted *PRIOR* to Robotics session:
(Please attach relevant investigations. If additional details/comments are needed, please attach)

Nature of the Safety Concern: ____

Brief description: ____

____

Severity (mild, moderate, or severe as per CTCAE v5): ______________________________

Was any intervention required?:	YES	NO	Details: ______________________
Was hospitalisation required?:	YES	NO	Details: ______________________
Did it limit instrumental ADLS?	YES	NO	Details: ______________________
Did it limit self-care ADLs?	YES	NO	Details: ______________________
Was it life-threatening?	YES	NO	Details: ______________________

CTCAE v5 Grade (circle): 1 2 3 4 5

Relationship to Robotics (circle): CERTAIN PROBABLE POSSIBLE UNLIKELY

Follow-up of any safety concerns noted *DURING* the *PREVIOUS* Robotics session:

Were there any safety concerns during the previous robotics session? YES NO

Nature of the Safety Concern: ____

Description: ____

____

Severity (mild, moderate, or severe as per CTCAE v5): ______________________________

Was any intervention required?:	YES	NO	Details: ______________________
Was hospitalisation required?:	YES	NO	Details: ______________________
Did it limit instrumental ADLS?	YES	NO	Details: ______________________
Did it limit self-care ADLs?	YES	NO	Details: ______________________
Was it life-threatening?	YES	NO	Details: ______________________

CTCAE v5 Grade (circle): 1 2 3 4 5

Causality (circle): CERTAIN PROBABLE POSSIBLE UNLIKELY

Supplementary File S1: Safety Checklist

Work-sheet

Participant Name: ______________________________ Date of Birth:___________________________

Today's Date: _________________________________ Session number: ________________________

Did the patient attend the previous prescribed session? YES NO

If "NO", reason(s) for non-attendance: ____

Was there a delay between the previous session and today's? YES NO

If "YES", reason(s) for delay: ____

Start time:______________

Did today's session need to be interrupted or stopped early? YES NO

If "YES", please circle: INTERRUPTION STOPPED EARLY

Reason: ____

Details of any safety concerns noted *<u>during</u>* today's Robotics session:

Were there any safety concerns during today's robotics session? YES NO

Nature of the Safety Concern: ____

Description: ____

____

Severity (mild, moderate, or severe as per CTCAE v5): ______________________________________

Was any intervention required?: YES NO Details: _________________________________

Was hospitalisation required?: YES NO Details: _________________________________

Interim CTCAE v5 Grade (circle): 1 2 3 4 5

Interim Causality (circle): CERTAIN PROBABLE POSSIBLE UNLIKELY

Date of next session: ________________________

Investigator Name: ________________________ **Signature**: ________________________

Notes:
