# Supplementary material for: Safety, Feasibility and Efficacy of Lokomat® and Armeo®Spring Training in Deconditioned Paediatric, Adolescent and Young Adult Cancer Patients
Source: Cancers (Basel). 2023 Feb 16;15(4):1250. doi: 10.3390/cancers15041250 (PMC9954270; doi:10.3390/cancers15041250)
Supplement: Supplementary file 1 [file cancers-15-01250-s001.zip › Supplementary File S2 robotics questionnaire.pdf]

Supplementary File S2: Robotics Questionnaire

Government of South Australia
SA Health

Women's & Children's Hospital

WOMEN'S AND CHILDREN'S HOSPITAL

Robotics Study Questionnaire

Participant Information

Name			Date of birth	
Date				
Treatment block	First ☐	Second ☐		

Ratings

Please tick one box for each row.	Strongly Disagree	Disagree	Neutral	Agree	Strongly Agree
**The sessions were fun.**	☐	☐	☐	☐	☐
Comments					
**I was comfortable during the treatment.**	☐	☐	☐	☐	☐
Comments					
**I liked coming for the sessions.**	☐	☐	☐	☐	☐
Comments					
**I feel I have improved during the sessions.**	☐	☐	☐	☐	☐
Comments					
**I would continue with sessions if they were available.**	☐	☐	☐	☐	☐
Comments					
**The frequency of sessions was appropriate.**	☐	☐	☐	☐	☐
Comments					
**The duration of the treatment block was appropriate.**	☐	☐	☐	☐	☐
Comments					
Overall Rating *(mark "X" on line)*	1		5		10

Questions

BEST PART ABOUT THIS TREATMENT

WORST PART ABOUT THIS TREATMENT

Additional comments
