# Supplementary material for: Safety, Feasibility and Efficacy of Lokomat® and Armeo®Spring Training in Deconditioned Paediatric, Adolescent and Young Adult Cancer Patients
Source: Cancers (Basel). 2023 Feb 16;15(4):1250. doi: 10.3390/cancers15041250 (PMC9954270; doi:10.3390/cancers15041250)

Supplementary File S3 – Outcome measures histograms

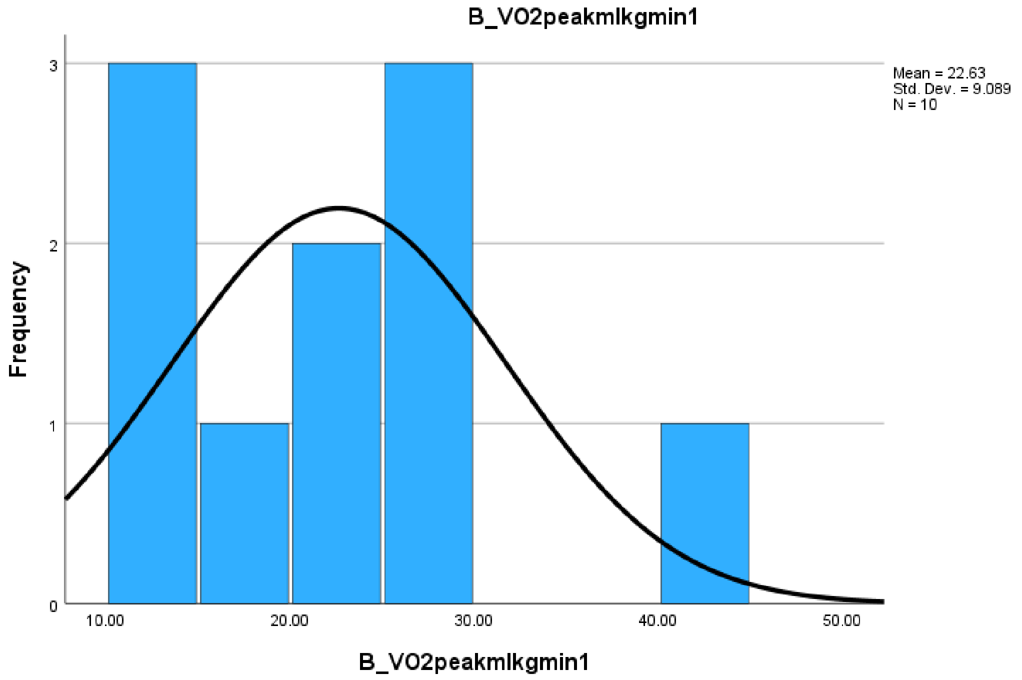

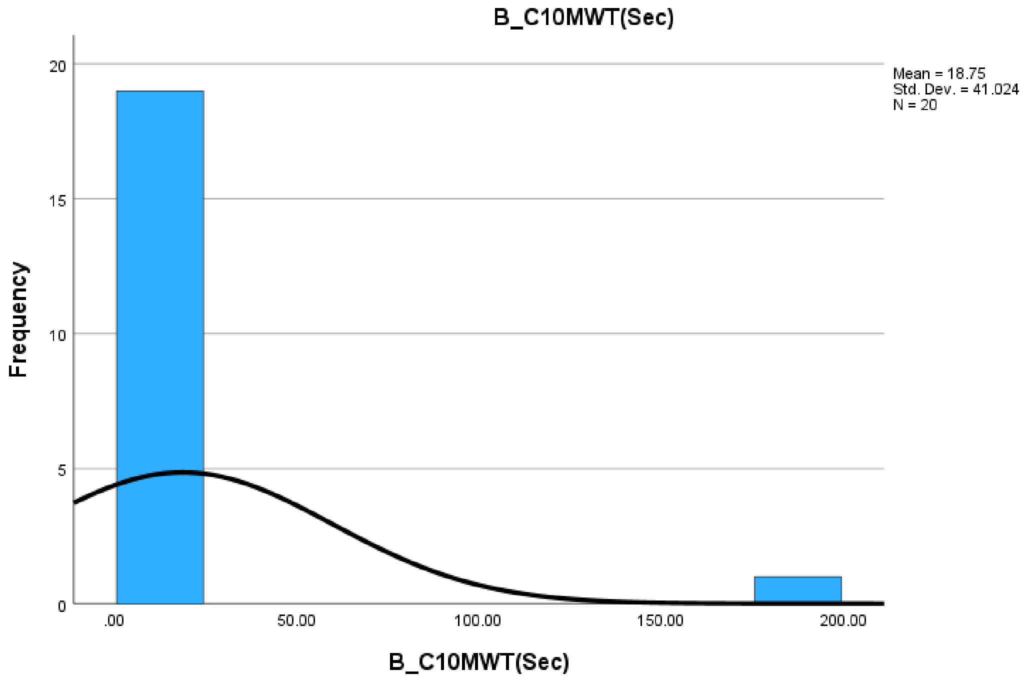

Supplementary File S3 – Outcome measures histograms

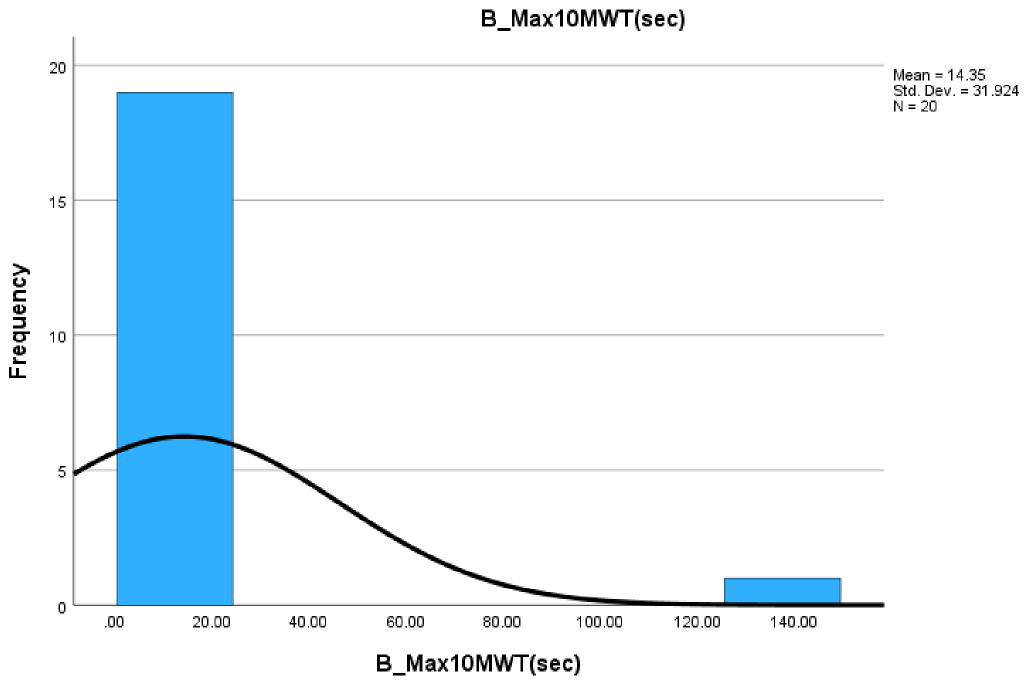

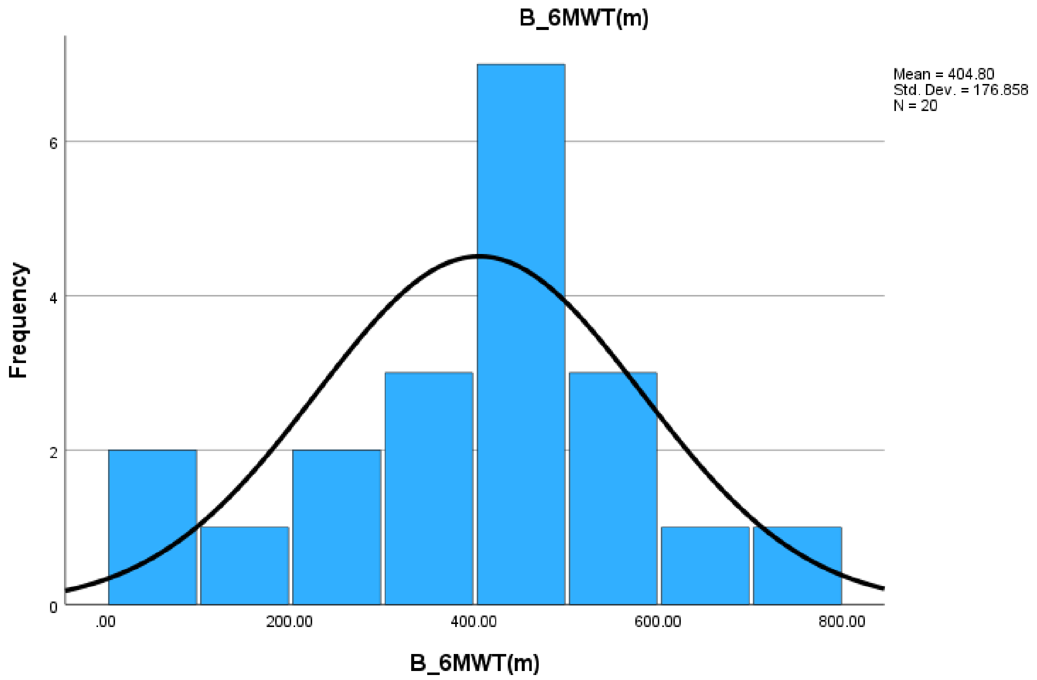

Supplementary File S3 – Outcome measures histograms

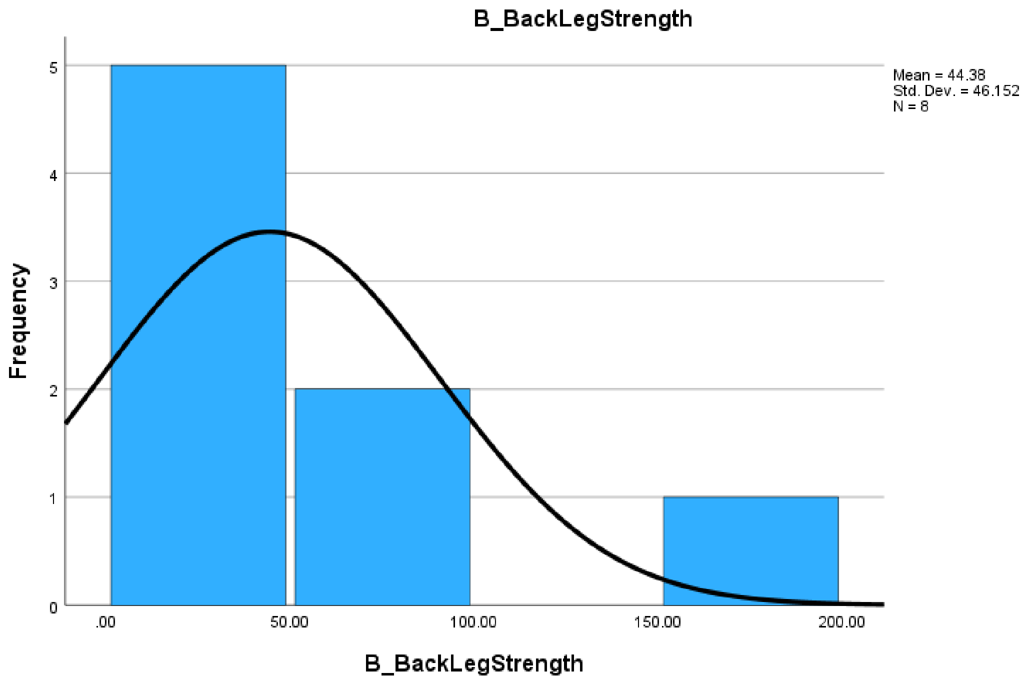

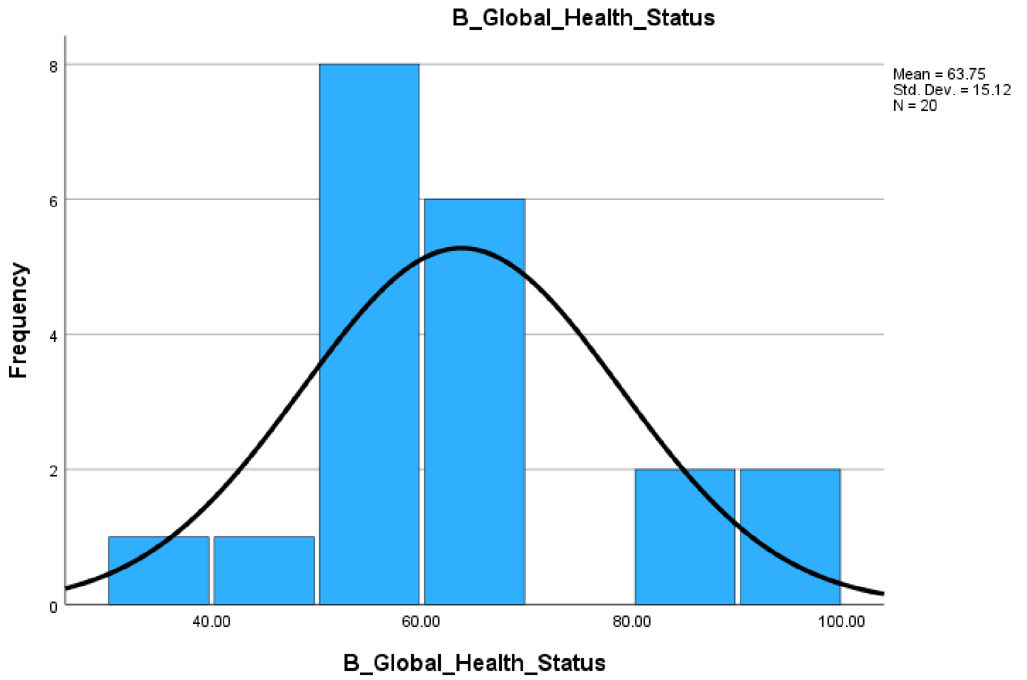

Supplementary File S3 – Outcome measures histograms

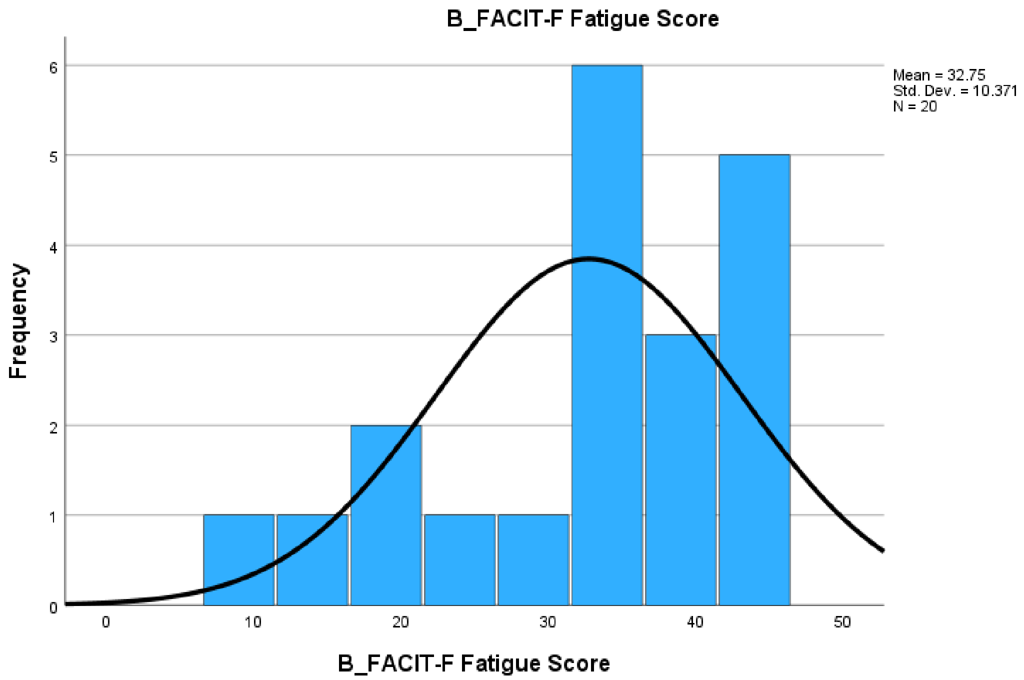

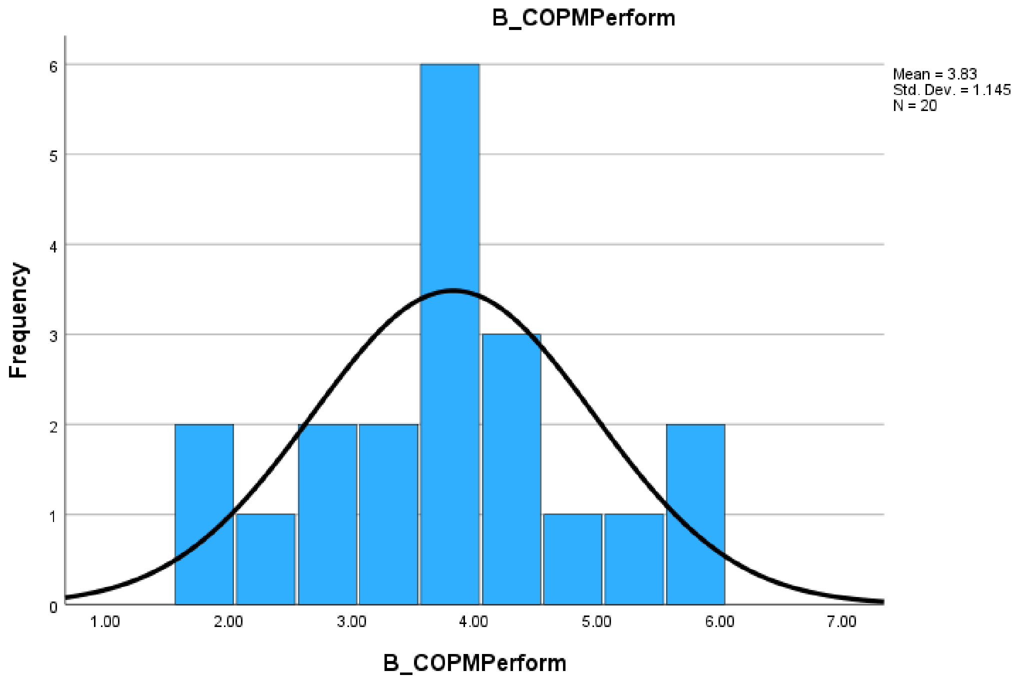

Supplementary File S3 – Outcome measures histograms

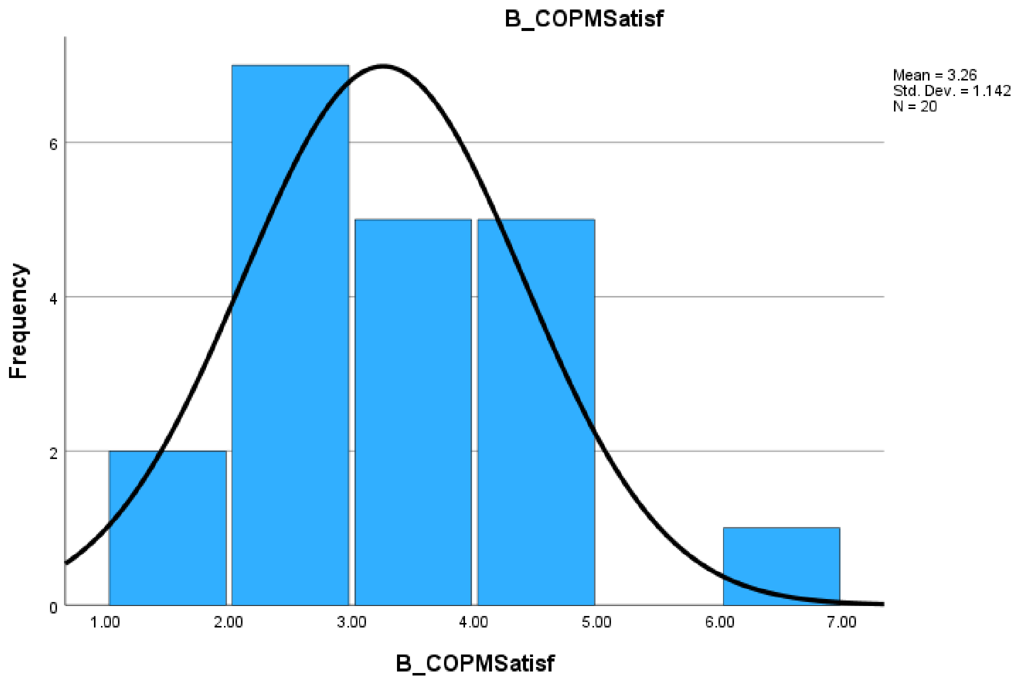

Supplement: Supplementary file 1 [file cancers-15-01250-s001.zip › Supplementary File S3 Outcome measures histograms.pdf]
