# Supplementary material for: Safety, Feasibility and Efficacy of Lokomat® and Armeo®Spring Training in Deconditioned Paediatric, Adolescent and Young Adult Cancer Patients
Source: Cancers (Basel). 2023 Feb 16;15(4):1250. doi: 10.3390/cancers15041250 (PMC9954270; doi:10.3390/cancers15041250)
Supplement: Supplementary file 1 [file cancers-15-01250-s001.zip › Supplementary File S4 Preliminary efficacy results Armeo docx.pdf]

Supplementary File S4: Preliminary efficacy results - Armeo®

Preliminary efficacy results - Armeo®				
**Outcome measure**	**Baseline all participants** mean SD n	**Baseline completers** mean SD n	**6 weeks** mean SD n	**Effect Size**
Box and block test (right arm)	45.3 (29.0) (n=3)	All completed	51.33 (33.8) (n=3)	0.47
Box and block test (left arm)	24.0 (29.7) (n=3)	All completed	29.3 (38.1) (n=3)	0.77
Back scratch (right arm)	21.5 (21.7) (n=3)	All completed	16.6 (16.3) (n=3)	0.62
Back scratch (left arm)	16.7 (16.3) (n=3)	All completed	15.3 (5.9) (n=3)	0.0
Maximal grip strength (right arm)	22.0 (10.0) (n=3)	All completed	22.3 (9.5) (n=3)	0.58
Maximal grip strength (left arm)	11.7 (7.6) (n=3)	All completed	14.0 (9.2) (n=3)	0.92
30 sec Arm Curl (right arm)	13.3 (6.5) (n=3)	All completed	16.0 (10.2) (n=3)	0.78
30 sec Arm Curl (left arm)	9.0 (10.8) (n=3)	All completed	10.3 (13.8) (n=3)	0.26
COPM Performance	5.1 (0.5) (n=3)	All completed	6.4 (2.3) (n=3)	0.62
COPM Satisfaction*	4.6 (0.9) (n=3)	All completed	6.4 (1.8) (n=3)	0.93
EORTCQLQC30**	66.7 (22.1) (n=3)	All completed	75.0 (14.4) (n=3)	0.31
FACIT-F***	40.7 (10.0) (n=3)	All completed	46.0 (4.0) (n=3)	0.62
GLTPAQ (LSI)****	21.3 (19.1) (n=3)	All completed	8.0 (13.9) (n=3)	0.78

* Canadian Occupational Performance Measure
**European Organisation for Research and Treatment of Cancer, Quality of Life Questionnaire, Global Health Status
*** The Functional Assessment of Chronic Illness Therapy – Fatigue Scale
**** Godin Leisure Time Physical Activity Questionnaire – Leisure Score Index
